# Health care system resilience - Evaluating the effect of the COVID-19 pandemic on emergency medical service demand in Germany: A case study from the city of Jena

**DOI:** 10.1371/journal.pone.0344992

**Published:** 2026-03-16

**Authors:** Jonas Brock, Harald Lux, Sebastian Lang, Johannes Winning, Arthur Gratzias, Jan-Christoph Lewejohann, Michael Bauer, Petra Dickmann

**Affiliations:** 1 WG Public Health, University Hospital Jena, Clinic for Anesthesiology and Intensive Care Medicine, Jena, Germany; 2 Leibniz Center for Photonics in Infection Research (LPI), Jena, Germany; 3 Thuringian Centre for Quality and Research in Emergency Medicine and Emergency Medical Services (ThuZenQ), Jena, Germany; 4 Clinic for Emergency Medicine, University Hospital Jena, Jena, Germany; 5 Department of Health and Nursing, Ernst Abbe University of Applied Sciences Jena, Jena, Germany; Jazan University College of Applied Medical Science, SAUDI ARABIA

## Abstract

**Background:**

The COVID-19 pandemic has impacted health systems globally, including emergency medical services (EMS). This study evaluates the absorptive and adaptive resilience of EMS in Jena, Germany, by analysing how the system managed significant pandemic-induced shocks to demand and operational workflows.

**Methods:**

We conducted an observational, retrospective analysis of EMS missions in Jena from January 2018 to December 2021. Data on mission volumes, patient demographics, health issues, and dispatch and treatment intervals were analysed. Lockdown periods were compared to corresponding pre-pandemic reference periods to determine significant differences.

**Results:**

During first and second lockdown EMS mission volume decreased by 16% and 10% respectively, when comparing with reference periods. Patient age increased, with a mean age of 62.2 years during the first lockdown versus 58.9 years in the reference period. There was a notable rise in on-site treatments without hospital transport. Cardiovascular cases decreased during lockdown periods, while occurrence of psychiatric patients increased. Scene times and handover times extended significantly, contributing to longer total mission times.

**Conclusions:**

The pandemic led to shift in demand patterns, characterized by reduced overall demand, extended mission times, and a change in treatment focus from cardiovascular to psychiatric and psychosocial issues, reflecting changes in healthcare-seeking behaviour and operational challenges. While regional studies have examined mission volumes, our study provides a higher-resolution analysis of the effects of the COVID-19 pandemic on pre-clinical EMS in Germany and provides important insights to be used for health care planning and policy decision-making.

## 1. Introduction

The COVID-19 pandemic has affected health systems worldwide by straining resources, overwhelming hospitals, and necessitating the implementation of various measures to control the spread of the virus. Lockdowns, social distancing, and other public health interventions were introduced to mitigate transmission, fundamentally altering daily life and healthcare delivery [1,2].

The healthcare system faced numerous challenges, including increased demand for critical care, shortages of medical supplies, and the need to protect healthcare workers from infection [1–3]. Elective procedures were postponed, routine medical visits declined, and the focus shifted towards managing COVID-19 patients. Amid these changes, pre-hospital emergency medical services (EMS) emerged as a crucial component of the healthcare system, providing essential care and transport for patients with urgent medical needs.

Pre-hospital emergency medical services play a vital role in the healthcare system, offering initial medical care and facilitating the transport of patients to appropriate healthcare facilities [4]. The demand for EMS is influenced by various factors, including public health measures, disease prevalence, and changes in healthcare-seeking behaviour. The COVID-19 pandemic has significantly impacted these factors, altering the volume and nature of emergency ambulance missions globally (e.g., Andrew et al., 2021; Dicker et al., 2020; Katayama et al., 2023).

Initial reports from several countries indicated a reduction in the number of emergency missions during the pandemic. For instance, a study in Italy reported a substantial decrease in ambulance dispatches during the lockdown period [5]. Similarly, research conducted in Australia observed a decline in EMS activations, attributed to public reluctance to seek hospital care during the pandemic [6]. However, studies from other regions presented mixed results, with some areas experiencing increased EMS missions, especially for severe cases [7,8].

Understanding the impact of COVID-19 on EMS is critical for health system resilience. Resilience in health systems refers to their ability to absorb, adapt, and transform when exposed to shocks such as a pandemic. It is crucial to understand how systems react when they are put under stress to improve preparedness and response strategies for future public health crises. Despite numerous reports and studies on the impact of COVID-19 on various aspects of healthcare, there are still few detailed analyses specifically examining emergency deployments and pre-hospital care in Germany [9].

This study aims to investigate the impact of the COVID-19 pandemic on EMS utilization in Jena, Germany, a city representative of medium-sized urban centres. By analysing EMS mission data from 2018 to 2021, the study seeks to provide insights that can be generalized to similar settings. The specific objectives and research questions of this study are as follows:

The specific objectives of this study are to:

(i) investigate the variation in the number of EMS missions before and during the COVID-19 pandemic, particularly during lockdown periods.
(ii) examine the demographic characteristics (age and gender) of patients requiring EMS services and how these characteristics changed during the pandemic.
(iii) categorise the types and severity of health issues addressed by EMS, focusing on shifts in the prevalence of specific health problems during the pandemic.
(iv) Analyse impact of COVID-19 on EMS time intervals

The results of this study will provide key information to develop new recommendations for preparedness in pre-hospital settings in Germany. Understanding these changes is crucial for optimizing emergency medical services and ensuring that the healthcare system can adequately respond to future public health crises. This study aims to fill the gap in the existing literature by providing a comprehensive analysis of the impact of COVID-19 on emergency medical services in Germany, thus contributing valuable insights for healthcare planning and policy decision-making.

## 2. Data & methods

### 2.1 Study design and ethics

This study is an observational, retrospective, descriptive analysis of EMS demand in Jena during the COVID-19 pandemic. Approval for the study was obtained from the administration of Jena University Hospital under registration number *2024–3457-Daten*. In the design of this study, we followed the guidelines for reporting observational studies as laid down in the Strengthening the Reporting of Observational Studies in Epidemiology (STROBE) statement [10,11].

All patient data used in this study were provided in a fully anonymized format to ensure patient privacy and confidentiality. Anonymization was performed by the EMS registry manager by removing all personal identifiers, including patient names, addresses, dates of birth, and other potentially identifying details. The ethics committee of Jena University Hospital specifically requested and supervised the anonymization process to guarantee compliance with data protection standards. Given the retrospective nature of the study and the complete anonymization of patient information, the ethics committee waived the requirement for obtaining individual patient consent, as no patient could be individually identified based on the data used in this research.

### 2.2 Study area

The study was conducted in the city of Jena, located in the federal state of Thuringia, Germany. Jena is a medium-sized city with a population of approximately 110,000 inhabitants. The city’s demographic and healthcare characteristics make it a suitable representative of medium-sized urban centres in Germany, providing valuable insights that can be generalised to similar settings. The pre-hospital emergency medical services in Jena are organized in a two-tiered system, which is typical for Germany following the ‘Franco-German modal’ [12,13]. The standard response to an emergency call is an ambulance staffed with paramedics. In cases of suspected critical conditions or the need for advanced medical interventions, an emergency physician is dispatched alongside the paramedic team.

### 2.3 Data

#### 2.2.1 Included missions & EMS protocols.

Data were collected on all EMS missions conducted in Jena during the study period from 1st January 2018–31st December 2021. For this, we received anonymized data from the regional EMS registry at the Centre for Disease Prevention and Emergency Medicine in the city of Jena. Each EMS mission is documented using standardized digital PDF protocols, which are filled out by personnel on mobile tablets using electronic pens. This ensures a consistent digital format and eliminates errors associated with manual transcription or handwriting recognition. We utilized an automated extraction script to transfer relevant data fields from the anonymized PDF files into a structured CSV dataset. This automated process ensured that missing values in the final dataset strictly corresponded to fields left blank in the original source protocols rather than losses during data transfer. To validate the extraction, a random audit of 5% of the missions was performed, comparing the original digital protocols against the extracted records to ensure a zero-error rate in the transfer process. The data was provided on 15^th^ July 2024. In total, 51,641 missions were conducted in the research area and are included in this study. Each mission is documented with an EMS protocol, capturing various key variables including patient age, sex, NACA score indicating severity, main health issue, proportion of on-site treatments without transport to the hospital, as well as several mission time intervals.

#### 2.2.2 EMS time definitions.

The time interval definitions used in this study are based on previous studies and are briefly described [14–16]. The response time interval was defined as the time from the incoming call taken by the EMS dispatcher to the time the ambulance stopped at the patient’s location. The scene time interval was defined as the time from when the ambulance stopped at the scene to the time it departed the scene. The transport time interval was defined as the time from when the ambulance departed the scene to when it arrived at the emergency department (ED). The handover time interval was defined as the time from when the ambulance arrived at the ED to when the patient was formally handed over to the hospital staff. The total mission time was defined as the overall time from when the emergency call was taken to when the ambulance returned to the base or when the ambulance reported being available for a new mission.

#### 2.2.3 Health issue categorisation.

The clinical diagnoses made by paramedics or physicians, also known as clinical impressions, were grouped into generic categories for this study to enable an overarching descriptive analysis of main disease groups. Over 400 possible clinical impressions were consolidated into broader categories including abdomen, cardiovascular system, central nervous system, gynaecological, metabolic, paediatric, psychiatric, respiratory distress or failure, trauma, and other. The grouping of these clinical impressions is available in the supplement of this paper.

### 2.3 Statistical analysis

For each analysis part, we first provide a general overview of the four-year period (2018–2021) and then analyse the lockdown effect separately. The data were segmented into four groups: 1st Lockdown, 1st Reference, 2nd Lockdown, and 2nd Reference. Lockdown periods were defined based on government-imposed restrictions, while reference periods were the corresponding times in previous years (2018 & 2019) without lockdown measures. Specifically:

- 1st Lockdown Period: 1st April to 31st May 2020
- 1st Reference Period: 1st April to 31st May 2018–2019
- 2nd Lockdown Period: 1st November 2020–31st May 2021
- 2nd Reference Period: 1st November to 31st May 2018–2019

By utilising a two-year pre-pandemic average as a baseline, the reference periods naturally account for recurring seasonal patterns, such as annual influenza waves and weather-related health fluctuations. This approach ensured that the comparisons captured deviations specifically related to the pandemic response rather than typical seasonal variability.

**Fig 1:**
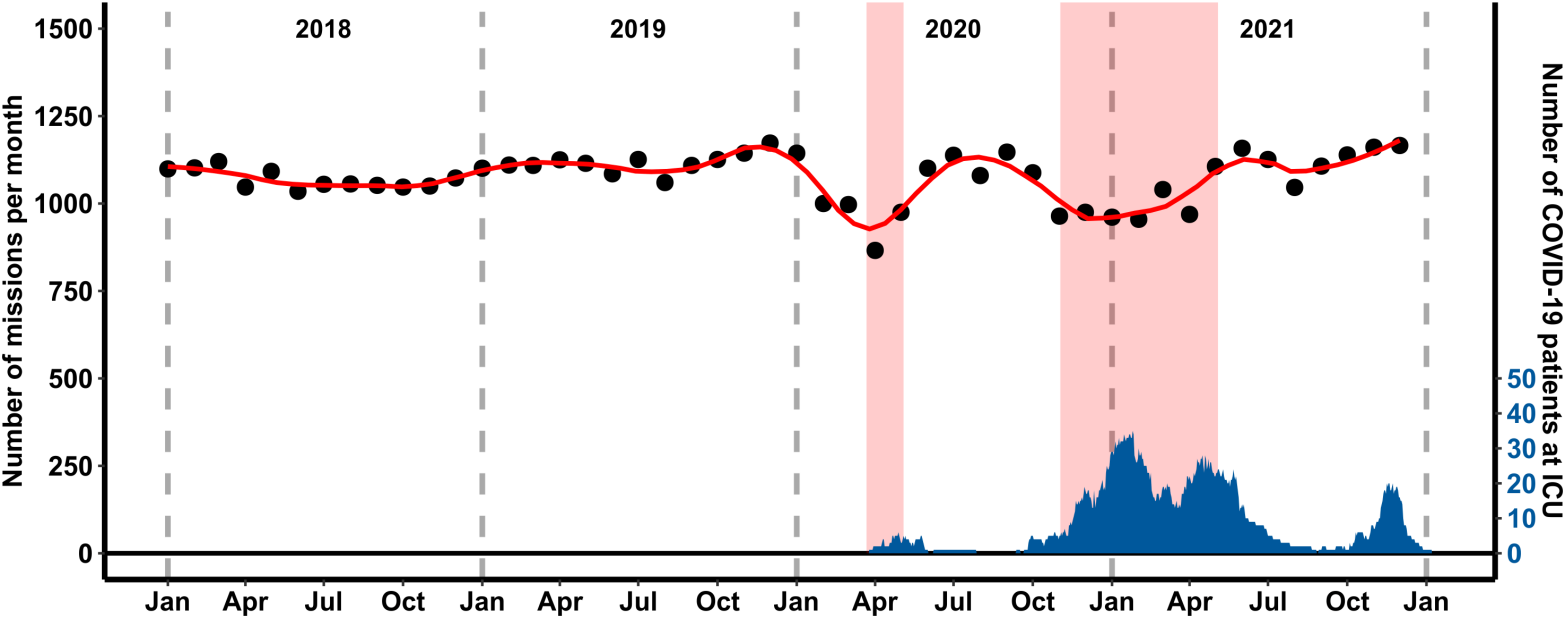
Monthly Number of EMS Missions and COVID-19 ICU Patients in Jena (2018−2021). The black dots represent the number of EMS missions per month, with the red line showing a smoothed trend. The blue area indicates the number of COVID-19 patients in intensive care units (ICU). Shaded red areas mark the first and second lockdown periods.

Continuous data are presented as means and ±SD. Categorical data are presented as percentages (%). The normality of continuous data was assessed using the Shapiro–Wilk test. Depending on the distributional properties of the data, appropriate statistical tests were applied. Specifically, for continuous variables, normally distributed data were compared using independent samples t-tests, whereas non-normally distributed data were analysed using the non-parametric Mann–Whitney U test. For categorical variables, differences between lockdown and reference periods were assessed with Pearson’s chi-square test, with Fisher’s exact test applied in cases of small sample sizes or when expected frequencies were below five. Given the multiple comparisons performed in this study, a Bonferroni correction was employed to adjust significance thresholds and control for type I error inflation. Adjusted two-tailed p-values of less than 0.05 were considered statistically significant. In addition to p-values, effect sizes were calculated to assess the magnitude of observed changes, using Cohen’s d for continuous data and Cramer’s V for categorical data. In addition, we applied the locally estimated scatterplot smoothing (LOESS) technique as described by [17] to visualise smoothed trend in mission volume and EMS time intervals. The smoothing parameter (*a*) was tailored to the specific data types – *a* of 0.2 was utilised for mission volume (Fig 1) to provide a robust overview of monthly trends, while *a* of 0.15 was used for mission time intervals.

## 3. Results

### 3.1. Number of EMS missions

The total number of EMS missions in Jena showed significant fluctuations over the study period (2018–2021). Fig 1 shows the monthly number of EMS missions (black dots) from January 2018 to December 2021 with a smoothed trend (red line) of the EMS missions over the entire study period. It is evident that the number of EMS missions per month remained relatively stable from 2018 to early 2020, with slight seasonal variations. However, a sharp decline in the number of missions is observed during the first lockdown period in 2020. Following this period, the number of missions begins to rise again but remains below pre-pandemic levels, with another decline observed during the second lockdown period.

The total number of EMS missions per year show a significant decrease of approximately 5.7% from 2019 (n = 13 231) to 2020 (n = 12 475) and a partial recovery of 3.7% from 2020 to 2021 (n = 12 934) (see Table 1). During the lockdown periods, there was an even more notable reduction in the number of EMS missions (see Table 2). For the first lockdown (April to May 2020), the mean number of missions was 1,842, a significant reduction compared to the 2,190 missions in the reference period (Incidence Rate Ratio [IRR] 0.84; 95% CI: 0.81–0.87; p < 0.001). Similarly, during the second lockdown (November 2020 to May 2021), the mean number of missions decreased to 6,970 from 7,740 in the reference period (IRR 0.90; 95% CI: 0.88–0.92; p < 0.001) (see Table 2).

**Table 1:** Characteristics of EMS Missions in Jena from 2018 to 2021.

	2018	2019	2020	2021
Patient characteristics				
Number of EMS missions	13 001	13 231	12 475	12 934
Age in years (mean ±SD)	58.9 ± 25.9	59.8 ± 26.2	61.3 ± 25.3	61.7 ± 25.5
Sex				
Female (%)	50.3	49.9	49.2	49.9
Male (%)	49.7	50.1	50.8	50.1
NACA score				
0 – no injury (%)	3.4	2.0	2.3	1.6
I – minor disturbance (%)	8	7.4	7.3	9.4
II – slight-moderate disturbance (%)	34.5	31.6	35.9	40.0
III – moderate-severe disturbance (%)	48.2	51.8	48.5	43.3
IV – serious incident (%)	2.9	3.7	3.6	3.6
V – acute danger (%)	1.3	1.3	1.1	0.8
VI – respiratory/cardiac arrest (%)	1.0	1.0	0.6	0.7
VII – death (%)	0.7	1.0	0.8	0.6
Prop. on-site treatments (%)	18.9	18.0	19.5	20.8
Time intervals as minutes				
Response time (mean)	8:19	8:34	8:34	8:41
Scene time (mean)	20:47	20:28	21:21	22:32
Transport time (mean)	11:24	11:35	11:46	11:46
Handover time (mean)	16:58	17:12	18:55	20:55
Total mission time (mean)	61:15	61:33	64:34	67:35
Physician at scene (%)	27.2	27.1	27.9	26.4

**Table 2:** Comparison of EMS missions and patient characteristics between lockdown and reference periods.

	1^st^ Lockdown			2^nd^ Lockdown		
	Reference	Lockdown	p- value/ES	Reference	Lockdown	p- value/ES
	1^st^ Apr to 31^st^ May 2018–2019	1^st^ Apr to 31^st^ May 2020		1^st^ Nov to 31^st^ May 2018–2019	1^st^ Nov 2020 to 31^st^ May 2021	
**Patient characteristics**						
Mean number of EMS missions	2 190	1 842	***<0.001 0.92***	7 740	6 970	***<0.001 0.41***
Age in years (mean ±SD)	58.9 ± 26.2	62.2 ± 24.6	***<0.001 0.32***	59.8 ± 26.1	63.1 ± 24.7	***<0.001 0.12***
Sex						
Female (%)	50.5	49.3	*0.470 0.01*	50.5	49.8	*0.443 0.01*
Male (%)	49.5	50.7	*0.470 0.01*	49.5	50.2	*0.443 0.01*
NACA score						
0 – no injury (%)	2.6	2.2	*0.934 0.06*	2.3	2.1	*0.832 0.09*
I – minor disturbance (%)	7.1	8.3	*0.657 0.06*	7.1	7.6	*0.662 0.09*
II – slight-moderate disturbance (%)	32.8	31.5	*0.805 0.06*	34.0	37.1	*0.094 0.09*
III – moderate-severe disturbance (%)	51.3	50.0	*0.775 0.06*	50.4	47.4	*0.132 0.09*
IV – serious incident (%)	3.5	5.1	*0.408 0.06*	3.0	3.9	*0.198 0.09*
V – acute danger (%)	0.7	1.1	*0.901 0.06*	1.3	0.9	*0.407 0.09*
VI – respiratory/cardiac arrest (%)	0.9	0.4	*0.672 0.06*	1.0	0.5	*0.267 0.09*
VII – death (%)	1.1	1.4	*0.906 0.06*	1.0	0.4	*0.141 0.09*
Prop. on-site treatments (%)	23.0	29.6	***<0.001 0.58***	24.6	27.5	***<0.001 0.42***
Time intervals as minutes						
Response time (mean)	8:18	8:57	*0.128 0.13*	8:25	8:46	*0.229 0.11*
Scene time (mean)	20:11	21:45	***<0.001 0.48***	20:48	22:40	***<0.001 0.51***
Transport time (mean)	11:24	11:39	*0.501 0.35*	11:28	11:41	*0.614 0.28*
Handover time (mean)	17:23	18.59	***<0.001 0.41***	16:59	20:34	***<0.001 0.37***
Total mission time (mean)	61:19	64:21	***<0.001 0.43***	61:33	67:53	***<0.001 0.42***
Physician at scene (%)	26.9	30.3%	***<0.001 0.51***	27.4	27.4	*0.825 0.12*

### 3.2. Patient characteristics

The average age of patients served by EMS increased over the study period, from 58.9 years in 2018 to 61.7 years in 2021 (see Table 1). This increase was particularly pronounced during the lockdown periods. During the first lockdown (April to May 2020), the mean age was 62.2 years, compared to 58.9 years in the reference period (April to May 2018–2019) (p < 0.001). Similarly, during the second lockdown (November 2020 to May 2021), the mean age increased to 63.1 years from 59.8 years in the reference period (November 2018 to May 2019) (p < 0.001) (see Table 2).

The gender distribution remained relatively stable throughout the period, with a slight predominance of male patients.

The NACA scores which indicate the severity of patients’ conditions showed minor variations, although no statistically significant differences between lockdown and reference periods for most categories were encountered.

### 3.3. On-site treatments & physician at scene

During the COVID-19 lockdowns a noticeable rise in the number of patients treated on-site without needing hospital transport was observed (see Table 2). In the first lockdown for example, in 29.6% of all mission only an on-site treatment was conducted, while this proportion was at 23% in the corresponding pre-pandemic period (p < 0.001). In the second lockdown a similar increase was observed, with on-site treatments accounting for 27.5% of missions, up from 24.6% in the reference period (p < 0.001).

The proportion of missions where a physician was present at the scene showed minor fluctuations but remained relatively consistent (see Table 1). In 2018, a physician was present in 27.2% of EMS missions, with a slight increase to 27.9% in 2020 and a decrease to 26.4% in 2021. During the first lockdown as listed in Table 2 the presence of physicians at the scene increased significantly to 30.3% from 26.9% in the reference period (p < 0.001). However, this trend did not continue into the second lockdown, where the presence of physicians remained at 27.4%, identical to the reference period.

### 3.4. Presenting symptom

The COVID-19 pandemic significantly impacted the types and frequencies of health issues addressed by EMS in Jena (Table 3). Cardiovascular problems, a major component of EMS missions, peaked at 13.4% in 2019 but slightly declined to 12.8% in 2021. This trend indicates a stable yet slightly reduced demand for emergency cardiovascular care during the pandemic.

**Table 3:** Distribution of Health Issues in EMS Missions in Jena (2018-2021).

	2018		2019		2020		2021	
Health Issue	Number	*%*	Number	*%*	Number	*%*	Number	%
Abdomen	802	*6.2*	923	*7.0*	890	*7.1*	973	*7.5*
Cardiovascular system	1627	*12.5*	1777	*13.4*	1634	*13.1*	1661	*12.8*
Central nervous system	480	*3.7*	465	*3.5*	433	*3.5*	433	*3.4*
Gynaecological	185	*1.4*	170	*1.3*	142	*1.1*	132	*1.0*
Metabolic	116	*0.9*	102	*0.8*	73	*0.6*	125	*1.0*
Paediatric	45	*0.4*	47	*0.4*	28	*0.2*	48	*0.4*
Psychiatric	664	*5.1*	633	*4.8*	609	*4.9*	670	*5.2*
Respiratory distress or failure	515	*4.0*	555	*4.2*	452	*3.6*	470	*3.6*
Trauma	2161	*16.6*	2386	*18.0*	2278	*18.3*	2214	*17.1*
Other	742	*5.7*	796	*6.0*	709	*5.7*	901	*7.0*
No information/misuse	5664	*43.6*	5377	*40.6*	5227	*41.9*	5307	*41.0*

Metabolic problems experienced a significant drop in 2020 to 0.6. Respiratory distress or failure cases also decreased from 4.2% in 2019 to 3.6% in both 2020 and 2021, which could be attributed to public health measures that reduced exposure to respiratory pathogens other than COVID-19.

Psychiatric cases saw a noticeable increase, rising to 5.2% in 2021. Meanwhile, trauma-related EMS missions, which constituted a substantial portion of missions, showed a minor decline from 18.3% in 2020 to 17.1% in 2021.

**Fig 2:**
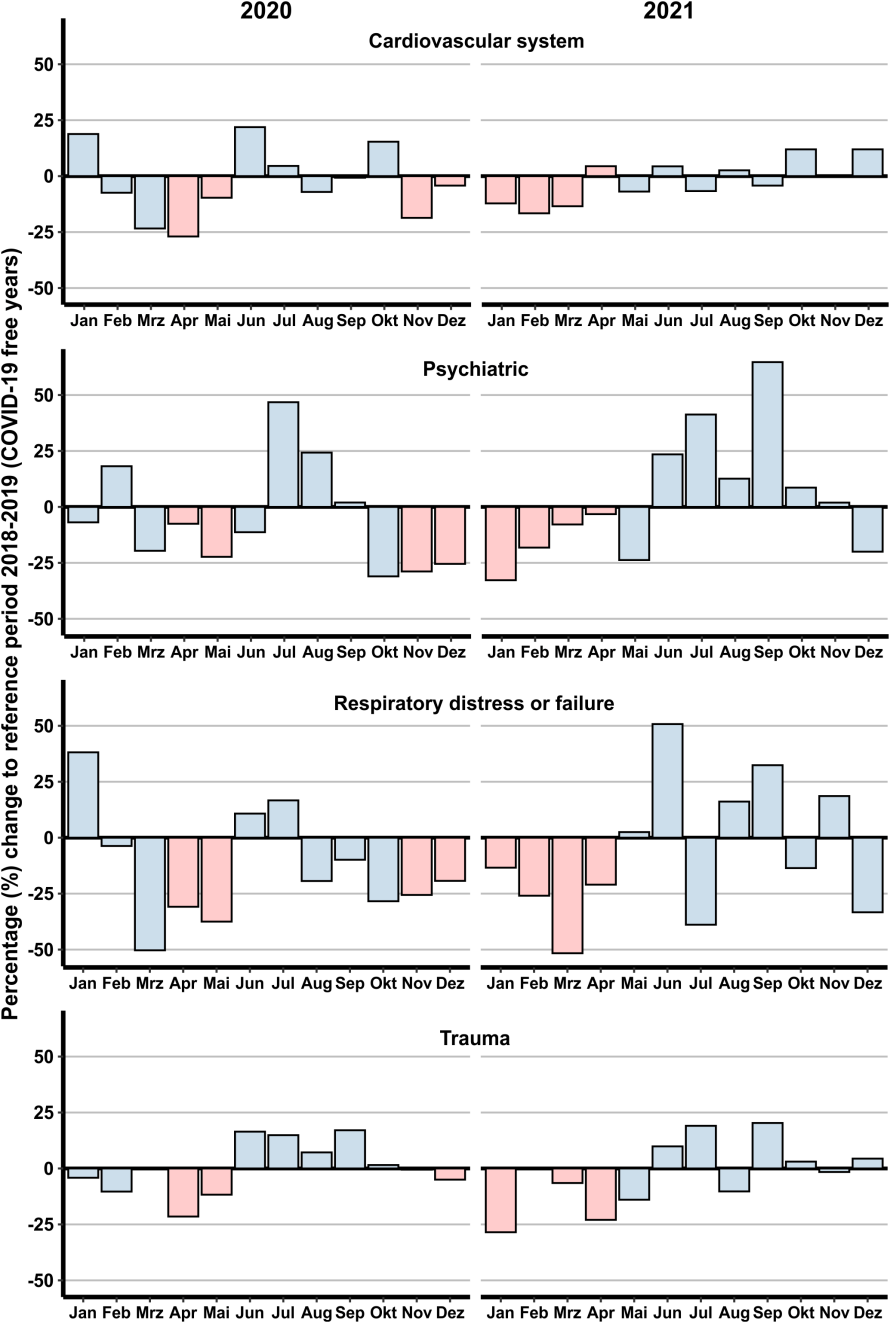
Monthly Percentage Changes in EMS missions for Selected Health Issues in Jena During the COVID-19 Pandemic. The figure shows the percentage change in emergency medical service missions for cardiovascular system issues, psychiatric problems, respiratory distress or failure, and trauma from the average values of 2018-2019 to those in 2020 and 2021. Red bars indicate months during which lockdown measures were in place.

Fig 2 illustrates the monthly differences in selected health problems, comparing the average values from 2018−2019 to those in 2020. The differences are expressed as percentages of increase or decrease, providing a clear visualisation of the impact of the COVID-19 pandemic and lockdown measures on EMS missions. Red bars in the figure represent the months during which lockdown measures were in place.

During the lockdown periods, cardiovascular issues showed significant fluctuations. In the early months of 2020 and 2021, there was a clear decrease in cases. Psychiatric health issues exhibited a pronounced increase during the summer months of 2020 and 2021, peaking notably higher in non-lockdown periods compared to the reference years. Respiratory distress or failure cases decreased sharply during the initial lockdown in early 2020 and 2021 but showed variable changes throughout the rest of both years. Trauma-related EMS missions saw a decrease during the lockdown months.

### 3.4. Mission frequency over a 24-hour period Intra-day mission distribution

**Fig 3:**
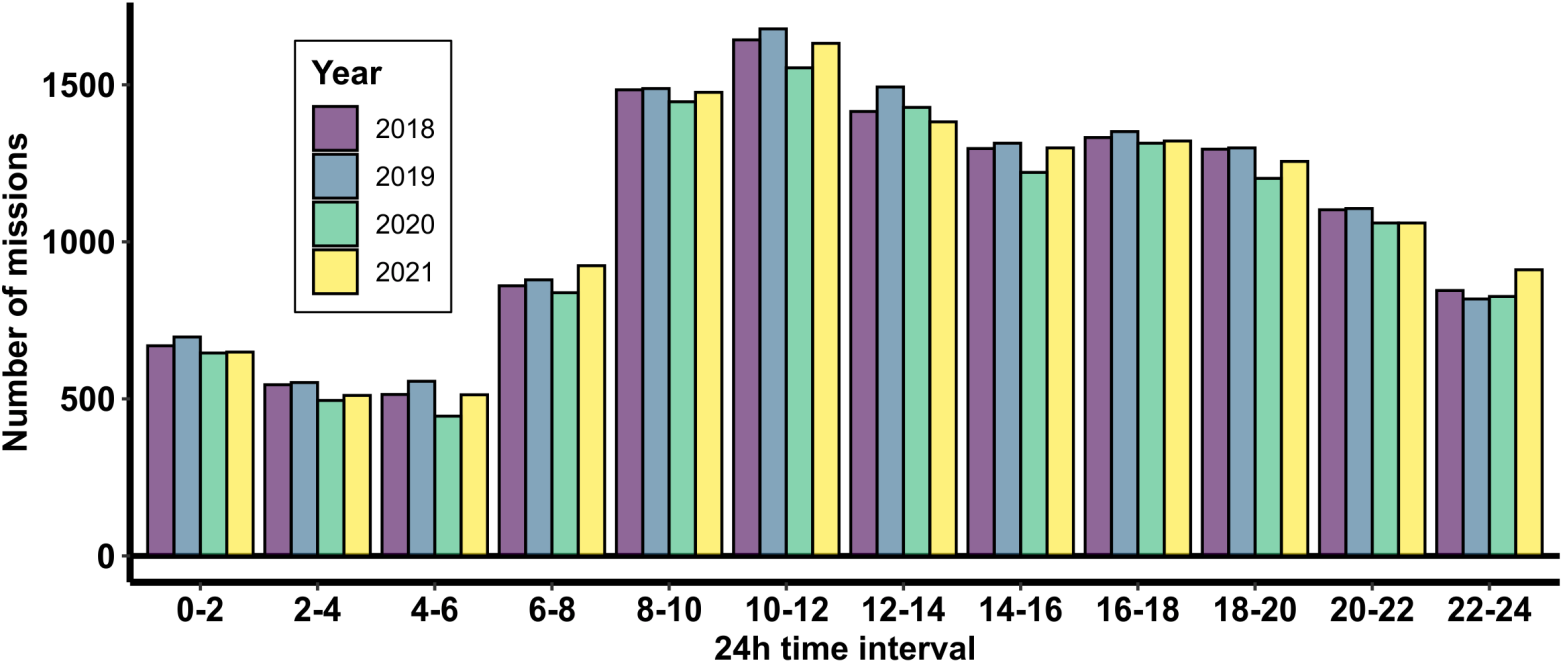
Distribution of the number of missions across 24-hour time intervals from 2018 to 2021.

The distribution of EMS missions over 24 hours from 2019−2021 is shown in Fig 3. The intra-day distribution pattern is similar for all years, with no exception during the COVID-19 affected years. During the early morning hours (0-6h), the number of missions is relatively followed by a significant increase in the number of missions during the morning hours, peaking between 10-12h. The mission counts remain relatively high during the afternoon hours (12-18h), followed by a gradual decline towards the evening/night.

### 3.5. EMS mission time intervals

**Fig 4:**
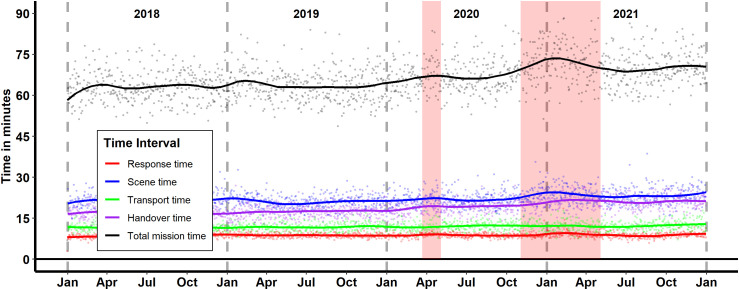
Trends in EMS Mission Time Intervals in Jena (2018−2021). The graph depicts the daily average times for various EMS intervals: response time (red), scene time (blue), transport time (green), handover time (purple), and total mission time (black). The shaded red areas represent the periods of the first and second COVID-19 lockdowns. The data points show individual mission times, and the solid lines represent smoothed trends using LOESS smoothing.

The total mission time, which encompasses all time intervals from the initial emergency call to the ambulance becoming available for a new mission, showed a noticeable increase during the pandemic. As depicted in Fig 4 and Table 1 the mean total mission time increased by more than 10% from 61:15 minutes in 2018–67:35 minutes in 2021. Especially during lockdown periods this increase was visible. Although response time and transport time remained largely unaffected, scene time and handover time experienced substantial delays (see Table 2). The mean scene time increased significantly during the first lockdown from 20:11 minutes to 21:45 minutes and during the second lockdown from 20:48 minutes to 22:40 minutes. Similarly, the mean handover time rose from 17:23 minutes to 18:59 minutes during the first lockdown and from 16:59 minutes to 20:34 minutes during the second lockdown. These increases in scene and handover times were the primary contributors to the overall extension of total mission time.

## 4. Discussion

The COVID-19 pandemic has posed challenges to healthcare systems worldwide, testing their resilience and ability to adapt to rapidly changing circumstances. In Jena, Germany, the demand for pre-hospital EMS was significantly affected by the pandemic, as evidenced by the substantial fluctuations in the volume and nature of EMS missions from 2018 to 2021. In our study, we examined how the pandemic influenced EMS demands.

### 4.1. Impact on EMS mission volume

The analysed data revealed a sharp decline in EMS missions during the first lockdown period in 2020, followed by a partial recovery and another decline during the second lockdown (see Fig 1). The initial reduction in EMS missions by ~16% aligns with reports from other countries such as Switzerland, Finland, Australia or New Zealand, where lockdown measures and public fear of infection led to a reluctance to seek pre-clinical emergency care [18,6,19,20]. While our dataset does not directly measure patient motivations, the observed 16% reduction in mission volume and the acute shift toward an older patient demographic provide empirical evidence of altered healthcare-seeking patterns. We contextualise these findings within existing international literature, which identifies public fear of infection and the ‘stay-at-home’ messaging of lockdowns as the primary drivers for the avoidance of emergency services during this period. The significant increase in the mean age of patients during lockdown periods, rising by 3.3 years during the first lockdown compared to its reference period, further supports the hypothesis of altered healthcare-seeking behaviour. While Germany’s population is aging overall, a shift of this magnitude in such a short timeframe cannot be attributed to secular demographic trends alone. Instead, it suggests a ‘selective avoidance’ pattern, where younger patients with potentially less severe conditions or higher levels of pandemic-related caution avoided calling EMS, while older patients with more acute or chronic care needs continued to require emergency services.

However, the gradual recovery in EMS mission volume post-lockdown (see Fig 1) indicates a return to more typical patterns of healthcare utilization and similar patterns have been observed in other countries [6]. The evident rebound could be attributed to the adaptation of the public to the new normal, increased confidence in the safety measures implemented by healthcare facilities, or a natural adjustment to the prolonged pandemic situation.

### 4.2. Changes in patient´s health issues

Cardiovascular problems remained a significant component of EMS missions but showed significant fluctuations. During the early months of 2020, there was an initial decrease in cardiovascular cases, likely due to patients’ reluctance to seek care during the lockdown. This was followed by an increase later in the year as restrictions eased and delayed cases surfaced (see Fig 2). The initial decrease in cardiovascular cases during the early lockdown period is supported by other studies in the field that have also documented a subsequent increase in cardiovascular cases (+18%) later in the year as restrictions eased [21]. Beyond care avoidance, alternative explanations for this decline should be considered. The pandemic-induced shift to remote work and the subsequent lockdowns may have resulted in a reduction of acute physical and psychological triggers, such as commuting-related stress or heavy occupational exertion. While reduced physical activity is a long-term risk factor, the immediate decrease in these environmental stressors might have contributed to a temporary decline in acute cardiovascular incidents during the early stages of the pandemic. Psychiatric issues, on the other hand, saw a notable increase, highlighting the mental health impact of the pandemic and associated lockdown measures. This has been described well in previous studies [20,22]. In terms of psychiatric cases and suicide diagnoses, numerous studies have documented the mental health burden imposed by the pandemic. While the current dataset did not permit a granular subdivision of psychiatric emergencies, the overall rise in demand aligns with reports of increased suicidal ideation, substance misuse, and acute anxiety disorders triggered by social isolation and economic uncertainty. Research highlighted that the pandemic exacerbated risk factors for suicidal behaviour, with several studies reporting increased incidences of suicidal ideation and attempts during this period [23]. A study focusing on youth suicide during the pandemic found that disruptions caused by COVID-19, such as changes to school and living environments, significantly impacted mental health and contributed to increased suicide rates among young people [24].

Respiratory distress or failure cases decreased during lockdown by partly more than 50% in comparison to the respective reference periods (see Fig 2). This reduction could be attributed to public health measures such as social distancing and mask-wearing, which likely reduced exposure to respiratory pathogens other than COVID-19. This observation aligns with findings from other studies that have documented similar trends. For instance, research conducted during the COVID-19 pandemic indicated significant declines in the incidence of various respiratory infections, including influenza and respiratory syncytial virus (RSV), which are common causes of respiratory distress and failure. The implementation of non-pharmaceutical interventions (NPIs), such as social distancing, mask-wearing, and improved hygiene practices, played a crucial role in reducing the transmission of these pathogens [25,26]. Other studies found that during the pandemic, influenza activity was at historically low levels, likely due to the widespread adoption of NPIs aimed at curbing the spread of COVID-19 [27]. This reduction in influenza and other respiratory infections can explain the observed decrease in EMS missions for respiratory distress or failure during lockdown periods.

Also, trauma-related EMS missions saw a decrease during the initial lockdown months, reflecting reduced outdoor activities and fewer accidents due to movement restrictions. However, as restrictions eased, there was a gradual increase in trauma cases, indicating a return to more typical activity levels and associated risks. This finding is well supported by previous studies [20,28,29].

### 4.3. On-site treatments & increased time intervals

Our analysis revealed a notable rise in the proportion of on-site treatments during the lockdowns, suggesting that EMS personnel were increasingly providing care without transporting patients to hospitals (Table 2). This shift may reflect efforts to minimize hospital overcrowding and reduce the risk of COVID-19 transmission. It also indicates the adaptability of EMS providers in managing patient care in situ, thereby alleviating some of the pressure on hospital emergency departments [30].

Additionally, the total mission time for EMS increased significantly during the pandemic, primarily due to extended scene and handover times (see Table 2 and Fig 4). The prolonged scene times could be linked to the complexity of managing patients in a pandemic context, including the need for additional protective measures and thorough on-site assessments. Paramedics were required to utilise enhanced Personal Protective Equipment (PPE) and perform more rigorous on-site infection screenings and disinfection procedures. These necessary safety protocols extended the duration of patient contact without hospital transport, contributing to the overall operational burden on the EMS system. The extended handover times likely reflect the increased burden on hospital staff and the implementation of stringent infection control protocols. This delay is commonly known as “ambulance offload delay” (AOD) and has been described elsewhere [14,16,31]. While this study did not directly measure the percentage reduction in daily EMS unit availability, the observed 10% increase in total mission time (rising from a mean of 61:15–67:35 minutes) represents a significant drain on system capacity. In a system operating at high utilisation rates, these cumulative delays effectively reduce the number of missions a single unit can complete per shift. The observed delays in our study emphasize the need for efficient coordination and communication between EMS and hospital teams to ensure timely patient care and resource management. To mitigate the observed ambulance offload delays in future crises, several practical policy interventions could be considered. The development of real-time, interoperable digital dashboards is essential to provide EMS dispatchers with live data on ED crowding. Also, the implementation of standardised triage protocols that bridge the pre-hospital and hospital settings can streamline the handover process, particularly when infection control measures are in place.

### 4.4. Limitations & future research

While this study provides comprehensive insights into the impact of COVID-19 on EMS demand in Jena, it is essential to acknowledge its limitations. The observational and retrospective nature of the study may introduce biases related to data recording and reporting. Additionally, our analysis did not explicitly model long-term secular trends, such as the gradual aging of the German population. While the suddenness of the age shifts observed during lockdowns points to a primary pandemic effect, the underlying demographic transition likely contributes to a higher baseline of EMS demand among older age groups over the four-year study period. Future studies should utilize age-standardization techniques to further isolate the impact of public health shocks from slow-moving demographic changes.

The findings from Jena, a medium-sized urban centre, may not be fully generalizable to larger cities or rural areas. Future research should aim to explore the long-term impacts of the pandemic on EMS systems and extend the analysis to diverse geographical settings. Comparative studies between different regions and countries could provide a broader understanding of the factors contributing to health system resilience and inform global health preparedness strategies.

While our study focused on the overall EMS mission volume and patient demographics, a comprehensive comparison of survival data across various patient categories during the pandemic would offer valuable insights into the broader impact of COVID-19 on patient outcomes. A systematic comparison of our EMS data with mortality tables for the study periods could provide a broader context for our findings, helping to identify whether the observed decrease in EMS missions corresponded with a general increase in mortality rates or if there were shifts in the causes of death. Recent studies have demonstrated significant reductions in live discharge rates, spontaneous circulatory recovery rates, and survival to hospital admission during the COVID-19 pandemic. For instance, a meta-analysis revealed that the survival to hospital admission rate decreased significantly during the pandemic, with an odds ratio (OR) of 0.63, indicating a substantial decline in successful pre-hospital interventions [32]. Additionally, there was an increased incidence of cardiac arrests at home, which likely reflects delays in or avoidance of seeking medical care due to pandemic-related fears [32].

Finally, this study is limited by the absence of patient outcome and mortality data. While our findings detail a reduction in EMS mission volume, we cannot conclude whether this led to increased community mortality or delayed care for critical conditions. Correlating these operational shifts with mortality tables remains a necessary step for future research to fully assess the impact of pandemic-induced service disruptions on public health outcomes.

### 4.5. Findings in the context of health system resillience

Framing the findings of this study within the health system resilience framework offers a structured overview of how EMS responds to pandemic-induced shocks. The component of awareness is highlighted by the significant decline in mission volumes, suggesting that public hesitation in seeking care stems from a lack of real-time surveillance and effective communication regarding EMS safety. While ICU data was integrated into public dashboards, a similar lack of transparency regarding EMS capacity limits hindered systemic awareness. Diversity and self-regulation were demonstrated through the increased rate of on-site treatments, which rose from 23% to nearly 30% during the first lockdown. These adaptations reduced pressure on hospital resources and ensured continued delivery through alternative pathways, reflecting the system’s adaptive capacity. However, the limited integration between EMS and hospitals, evidenced by significantly extended handover times, reveals a critical gap in interoperable data sharing. To improve transformative capacity, the system must move beyond reactive measures toward long-term innovations, such as enhanced digital infrastructure and stronger community-based care pathways, which strengthen overall operational resilience for future crises.

## 5. Conclusion

Our study provides valuable insights into the impact of COVID-19 on EMS demand and operations in Jena, Germany. The available data shows a substantial decrease in the number of EMS missions and prolonged time intervals after the beginning of the COVID-19 pandemic. The findings highlight the dynamic nature of healthcare systems and the importance of understanding how they respond to public health crises. These insights can inform future preparedness strategies and policy decisions, enhancing the resilience of emergency medical services and the broader healthcare system.

## References

[1] Filip R, Gheorghita Puscaselu R, Anchidin-Norocel L, Dimian M, Savage WK. Global challenges to public health care systems during the COVID-19 pandemic: a review of pandemic measures and problems. J Pers Med. 2022;12(8). 10.3390/jpm12081295 PMC9409667 36013244

[2] Rosenthal A, Waitzberg R. The challenges brought by the COVID-19 pandemic to health systems exposed pre-existing gaps. Health Policy Open. 2023;4:100088. doi: 10.1016/j.hpopen.2022.100088 36536931 PMC9753444

[3] Nana-Sinkam P, Kraschnewski J, Sacco R, Chavez J, Fouad M, Gal T, et al. Health disparities and equity in the era of COVID-19. J Clin Transl Sci. 2021;5(1):e99. doi: 10.1017/cts.2021.23 34192054 PMC8167251

[4] McIntosh BA, Hinds P, Giordano LM. The role of EMS systems in public health emergencies. Prehosp Disaster Med. 1997;12(1):30–5. doi: 10.1017/s1049023x00037183 10166372

[5] Valent F, Licata S. Emergency medical services calls during Italy’s COVID-19 lockdown. Ann Emerg Med. 2020;76(6):812. 33222791 10.1016/j.annemergmed.2020.06.036 PMC7306703

[6] Andrew E, Nehme Z, Stephenson M, Walker T, Smith K. The Impact of the COVID-19 Pandemic on Demand for Emergency Ambulances in Victoria, Australia. Prehospital Emergency Care. 2021;26(1):23–9. 34152925 10.1080/10903127.2021.1944409

[7] Moskatel LS, Slusky DJG. The impact of COVID-19 incidence on emergency medical services utilization. J Emerg Med. 2023;65(2):e111-8. 10.1016/j.jemermed.2023.04.017 PMC10129907 37460386

[8] Çavuş K, Akbulut M, Kaya AA. The impact of the COVID-19 pandemic on pre-hospital emergency medical services: The impact of the Covid-19 pandemic on pre-hospital services. Disaster Med Public Health Prep. 2024;18. 10.1017/dmp.2024.23 38374588

[9] Dax F, Waibel M, Kneißl K, Prückner S, Lazarovici M, Hoffmann F, et al. Analyzing emergency call volume, call durations, and unanswered calls during the first two waves of the COVID-19 pandemic compared to 2019: An observational study of routine data from seven bavarian dispatch centres. Heliyon. 2024;10(3):e24839. 10.1016/j.heliyon.2024.e24839 PMC10850415 38333836

[10] Vandenbroucke JP, von Elm E, Altman DG, Gøtzsche PC, Mulrow CD, Pocock SJ, et al. Strengthening the Reporting of Observational Studies in Epidemiology (STROBE): explanation and elaboration. PLoS Med. 2007;4(10):e297. doi: 10.1371/journal.pmed.0040297 17941715 PMC2020496

[11] Elm E, von E, Altman DG, Egger M, Pocock SJ, Gøtzsche PC, et al. Strengthening the reporting of observational studies in epidemiology (STROBE) statement: guidelines for reporting observational studies. BMJ: British Medical Journal. 2007;335(7624):806. 17947786 10.1136/bmj.39335.541782.AD PMC2034723

[12] Dick WF. Anglo-American vs. Franco-German emergency medical services system. Prehosp Disaster Med. 2003;18(1):29–35; discussion 35-7. doi: 10.1017/s1049023x00000650 14694898

[13] Hegenberg K, Althammer A, Gehring C, Prueckner S, Trentzsch H. Pre-Hospital Emergency Medical Services Utilization Amid COVID-19 in 2020: Descriptive Study Based on Routinely Collected Dispatch Data in Bavaria, Germany. Healthcare. 2023;11(14). 10.3390/healthcare11141983 PMC10379196 37510425

[14] Cone DC, Davidson SJ, Nquyen Q. A time-motion study of the emergency medical services turnaround interval. Ann Emerg Med. 1998;31(2):241–6. 10.1016/S0196-0644(98)70314-2 28139993

[15] Spaite DW, Valenzuela TD, Meislin HW, Criss EA, Hinsberg P. Prospective validation of a new model for evaluating emergency medical services systems by in-field observation of specific time intervals in prehospital care. Ann Emerg Med. 1993;22(4):638–45. doi: 10.1016/s0196-0644(05)81840-2 8457088

[16] Park YJ, Song KJ, Hong KJ, Park JH, Kim TH, Kim YS, et al. The Impact of the COVID-19 Outbreak on Emergency Medical Service: An Analysis of Patient Transportations and Time Intervals. J Korean Med Sci. 2023;38(42). 10.3346/jkms.2023.38.e317 PMC10615634 37904654

[17] Cleveland WS. Lowess: A program for smoothing scatterplots by robust locally weighted regression. Am Stat. 1981;35(1):54-.

[18] Dicker B, Swain A, Todd VF, Tunnage B, McConachy E, Drake H, et al. Changes in demand for emergency ambulances during a nationwide lockdown that resulted in elimination of COVID-19: an observational study from New Zealand. BMJ Open. 2020;10(12):e044726. doi: 10.1136/bmjopen-2020-044726 33361171 PMC7759754

[19] Laukkanen L, Lahtinen S, Liisanantti J, Kaakinen T, Ehrola A, Raatiniemi L. Early impact of the COVID-19 pandemic and social restrictions on ambulance missions. Eur J Public Health. 2021;31(5):1090–5. doi: 10.1093/eurpub/ckab065 33856015 PMC8083286

[20] Vuilleumier S, Spichiger T, Dénéréaz S, Fiorentino A. Not only COVID-19 disease impacts ambulance emergency demands but also lockdowns and quarantines. BMC Emerg Med. 2023;23(1):4. doi: 10.1186/s12873-023-00772-3 36635638 PMC9836922

[21] Goldberg SA, Cash RE, Peters G, Weiner SG, Greenough PG, Seethala R. The impact of COVID-19 on statewide EMS use for cardiac emergencies and stroke in Massachusetts. J Am Coll Emerg Physicians Open. 2021;2(1):e12351. doi: 10.1002/emp2.12351 33532755 PMC7823089

[22] Ferwana I, Varshney LR. The impact of COVID-19 lockdowns on mental health patient populations in the United States. Sci Rep. 2024;14(1):5689. doi: 10.1038/s41598-024-55879-9 38454064 PMC10920688

[23] Barlattani T, D’Amelio C, Capelli F, Mantenuto S, Rossi R, Socci V. Suicide and COVID-19: a rapid scoping review. Ann Gen Psychiatry. 2023;22(1):1–48. 36932453 10.1186/s12991-023-00441-6 PMC10020759

[24] Schnitzer PG, Dykstra H, Collier A. The COVID-19 Pandemic and Youth Suicide: 2020–2021. Pediatrics. 2023;151(3). 10.1542/peds.2022-058716 36789553

[25] Bhardwaj S, Choudhary ML, Chadha MS, Kinikar A, Bavdekar A, Gujar N, et al. Resurgence of respiratory syncytial virus infection during COVID-19 pandemic in Pune, India. BMC Infect Dis. 2024;24(1):586. doi: 10.1186/s12879-024-09426-6 38877428 PMC11177433

[26] Wagatsuma K, Koolhof IS, Shobugawa Y, Saito R. Decreased human respiratory syncytial virus activity during the COVID-19 pandemic in Japan: an ecological time-series analysis. BMC Infect Dis. 2021;21(1):734. doi: 10.1186/s12879-021-06461-5 34344351 PMC8329631

[27] Takeuchi H, Kawashima R. Disappearance and re-emergence of influenza during the COVID-19 pandemic: association with infection control measures. Viruses. 2023;15(1). 10.3390/v15010223 PMC9862942 36680263

[28] Azbel M, Heinänen M, Lääperi M, Kuisma M. Effects of the COVID-19 pandemic on trauma-related emergency medical service calls: a retrospective cohort study. BMC Emerg Med. 2021;21(1):102. doi: 10.1186/s12873-021-00495-3 34503453 PMC8426589

[29] Pettke A, Stassen W, Laflamme L, Wallis LA, Hasselberg M. Changes in trauma-related emergency medical services during the COVID-19 lockdown in the Western Cape, South Africa. BMC Emerg Med. 2023;23(1):72. doi: 10.1186/s12873-023-00840-8 37370047 PMC10304331

[30] Satty T, Ramgopal S, Elmer J, Mosesso VN, Martin-Gill C. EMS responses and non-transports during the COVID-19 pandemic. Am J Emerg Med. 2021;42:1–8. 33429185 10.1016/j.ajem.2020.12.078 PMC7836527

[31] Eckstein M, Isaacs SM, Slovis CM, Kaufman BJ, Loflin JR, O’Connor RE, et al. Facilitating EMS turnaround intervals at hospitals in the face of receiving facility overcrowding. Prehosp Emerg Care. 2005;9(3):267–75. doi: 10.1080/10903120590962102 16147474

[32] Kim JH, Ahn C, Park Y, Won M. Comparison of out-of-hospital cardiac arrests during the COVID-19 pandemic with those before the pandemic: an updated systematic review and meta-analysis. Front Public Health. 2023;11:1180511. 37234770 10.3389/fpubh.2023.1180511 PMC10208072

